# Altered vaginal microbiome and relative co-abundance network in pregnant women with penicillin allergy

**DOI:** 10.1186/s13223-020-00475-5

**Published:** 2020-09-09

**Authors:** Xiaohua Li, Jun Luo, Chuan Nie, Qingxia Li, Xiaofeng Sun, Hongping Li, Yong Zhang

**Affiliations:** 1 Chengdu Women’s and Children’s Central Hospital, School of Medicine, University of Electronic Science and Technology of China, Chengdu, 610000 China; 2 Shenzhen Baoan Women’s and Children’s Hospital, Jinan University, Shenzhen, 518000 China; 3 Guangdong Women and Children Hospital, No. 13, Guangyuan West Road, Yuexiu District, Guangzhou, 510000 China; 4 Shenzhen Children’s Hospital, Shenzhen, 518000 China

**Keywords:** Penicillin allergy, Vaginal microbiome, Co-abundance network, Neonatal health, 16S rRNA sequencing

## Abstract

**Background:**

Penicillin allergy is frequently reported in adults and children. Recent studies suggest that microbiota plays a key role in the development and progression of allergy. In this study, the relationship between vaginal microbiome and pregnant women with penicillin allergy was investigated.

**Methods:**

Vaginal samples before labor from 12 pregnant women with penicillin allergy and 15 non-allergic pregnant women were collected. Bacterial community structure of all study subjects and the discrepancies between the two groups were analyzed using 16S rRNA sequencing based on Illumina Hiseq 2500 platform.

**Results:**

The abundant phyla among all participants were *Firmicutes, Actinobacteria* and *Bacteroidetes*. The predominant genus was *Lactobacillus*. Compared to non-allergic pregnant women, *Actinobacteria, Coriobacteriaceae*, *Lachnospiraceae*, *Paraprevotella* and *Anoxybacillus* significantly decreased, whereas *Deltaproteobacteria*, *Peptostreptococcaceae*, *Enterococcus* and *Megamonas* were more abundant in penicillin allergic women. Additionally, obvious discrepancies were observed in the co-abundance network at the genus level between the two groups.

**Conclusions:**

There were differences in the microbial community structure and composition of reproduction tract between penicillin allergic and non-allergic pregnant women. These shifts may be related to maternal and neonatal health.

## Background

Antibiotic allergy appears to be with a prevalence as high as 10% among the general population according to self reports [33]. Its incidence continues to increase in recent years and in industrialized countries [34]. As the most commonly reported antibiotic allergy, penicillin allergy is prevalent in the United States with the prevalence about 8–10% [3, 34]. Prior data suggests that pregnant women with penicillin allergy have significantly higher cesarean section rate, and are associated with more hospital care utilization and additional morbidity [13]. It demonstrates that pregnant women with penicillin allergy would have an important impact on maternal and neonatal health. Penicillin allergy is also more prevalent in families with allergic history as children would have a higher chance to develop allergy if parents have allergy [4, 16, 25]. Up to 10% of the US children is labeled as penicillin allergy [27].

Moreover, antibiotic allergy results in life-threatening anaphylactic reactions in affected people and leads to significant morbidity, quality of life impairment, and healthcare costs [9]. That raises the intriguing question of the mechanism under the pathogenesis of allergy. Growing studies demonstrate that genetic predisposition, the route of allergic sensitization, timing and dose of allergen exposure, as well as gut microbiome are related to allergy [20, 28, 31, 41, 44]. Thanks to the rapid development of NGS sequencing technology, increasing attentions have been paid to gut microbiome. More and more studies find that gut microbiome plays an important role in the development and progression of allergic diseases [7, 32, 41]. Particularly, infants with food allergy have different gut microbiota compared with healthy infants [24, 36]. It is increasingly clear that the maternal microbiome during pregnancy has a key role in preventing allergy-prone immune phenotype and influences the immune system of the offspring [45]. The initial bacterial colonizers of neonatal gut are acquired from the maternal vaginal microbiota during natural delivery, whereas cesarean-born infants are first exposed to the skin microbiome of parents and health providers [6, 35].

Thus, we hypothesized that a different community of vaginal microbiome existed in the pregnant women with penicillin allergy, and it would be transmitted to newborns through vaginal delivery, which would make the newborns have higher risk to penicillin allergy, and this would partly explain the familial aggregation of penicillin allergy. In this study, we recruited 12 healthy pregnant women with penicillin allergy, compared their vaginal microbiome before labor to that of non-allergic women. We aimed to elucidate the pattern change of vaginal microbiome in pregnant women with penicillin allergy.

## Methods

### Recruitment of subjects

Twenty-seven reproductive-ages and asymptomatic pregnant women with gestational age > 37 weeks were enrolled (12 women with penicillin allergy (AG) and 15 non-allergic controls (NG)), with the following exclusion criteria: self-reported allergic rhinitis, atopic eczema and asthma; use of probiotics, prebiotics or synbiotics in the previous month; bacterial vaginosis and known active bacterial, fungal, and/or viral infection. Moreover, all participants were with newborns whose Apgar scores were 10 in 1 min after birth and without clinical signs of vaginal diseases. Penicillin allergy was diagnosed and assessed according to the British Society for Allergy and Clinical Immunology guidelines [26]. All recruited participants were from Shenzhen Baoan Women’s and Children’s Hospital, between June 2017 and October 2017. Penicillin allergic women’s diagnosis were established by clinical history or symptoms within 3 years, while control group were those pregnant women who did not have allergic manifestations. All subjects’ allergy statuses were confirmed with skin prick tests twice before their recruitment using CONBA penicillin for skin test reagents (produced by Zhejiang Jinhua CONBA Bio-pharmaceutical Co. LTD). Following a 20 min waiting period after skin pricks, positive reaction was reported with a wheal of 10 mm or more in diameter with surrounding flare greater than the wheal. The study protocol was approved by the Ethics Committee of Shenzhen Baoan Women’s and Children’s Hospital (No. QKTLL-2017-05-04). Informed written consents were obtained from all participants prior to enrollment. Additionally, clinical characteristics of all study participants were extracted from the health records.

### Sample collection and DNA extraction, PCR and sequencing

Vaginal samples were collected by midwives using sterile swabs when the participating women presented to the labor ward and before they underwent any examinations. Sterile swabs were placed carefully on the vaginal sidewall about halfway between the introitus and the cervix, followed the instructions reported previously [39]. Three swabs were obtained for every sample to make sure DNA content. All samples were transferred immediately to the laboratory and stored at − 80 °C until extraction. DNA was extracted from vaginal swabs using QIAamp DNA Mini kit according to the manufacturer’s instructions. The amount of DNA was determined using NanoDrop ND-1000 spectrophotometer (Thermo Electron Corcopration). All DNA was stored at − 20 °C before further analysis.

The bacterial genomic DNA was amplified with the forward primer 338F (5′-ACTCCTACGGGAGGCAGCAG-3′) and reverse primer 806R (5′-GGACTACHVGGGTWTCTAAT-3′) specific for V3–V4 hyper-variable regions of 16S rRNA gene. PCR amplification was performed in a volume of 50 μl, containing 25 μl 2× Premix Taq (Takara Biotechnology, Dalian Co. Ltd., China), 2 μl of each 10 mM primer, and 3 μl DNA template. The integrity and size of amplification products were visualized and checked with 1.0% agarose gel electrophoresis. Equimolar concentration of 27 samples were pooled and sequenced on an Illumina Hiseq 2500 machine with 2× 250 flow cell.

### Bioinformatics and statistical analysis

The resulting sequencing reads were firstly separated according to barcode and primer sequences using custom Perl scripts. The sequencing reads were paired-end joined, filtered based on the quality score using Mothur (version 1.39.5) software [37], and culled out the sequences with chimeras detected by UCHIME [15]. Remained sequences were clustered into operational taxonomic units at 97% similarity. Resulting operational taxonomic units were taxonomically classified by Ribosomal Database Project (RDP) Naïve Bayesian Classifier with training set (version 16) [47]. Alpha diversity, which represented using Shannon, Simpson indices and observed OTU (operational taxonomic unit) number, and weighted UniFrac distances were calculated and compared between pregnant women with and without penicillin allergy.

Comparisons of demographic and clinical variables of this study cohort were performed in R software with 0.05 as the defining p-value indicating statistically significance using Chi square and *t*-tests. Chi square test was used to evaluate the difference of categorical variables. Continuous variables were presented as mean ± standard deviation (SD). Their differences were investigated using unpaired *t*-test. Principle coordinates analysis (PCoA) was performed using vegan package in R software based on Bray–Curtis similarity.

LEfSe (Linear Discriminant Analysis Effect Size) analysis [38] was used to identify microbial residents enriched in penicillin allergic or non-allergic pregnant women with LDA score bigger than 2 and *P* value < 0.05. Pearson’s correlation with coefficient > 0.45 or < − 0.35 was selected for co-abundance network construction as described before [46]. The co-abundance microbial networks of AG and NG groups were constructed using Cytoscape software [40].

## Results

### Demographic data

The vaginal samples were collected from 12 penicillin allergic and 15 non-allergic pregnant women when they presented to the labor ward before any examinations. The demographic and clinical characteristics of all participants and their newborns were detailed in Table 1. All women in this study were Han Chinese with pregnant ages ranging from 27 to 39 years old (average: 32.3 years). Five subjects in AG group had pregnancy complications (3 with gestational diabetes, 1 was a *Hepatitis B* carrier, and 1 with front placenta). Meanwhile, 5 subjects in NG group had complications (2 with thalassemia, 1 with gestational diabetes, 1 with uterine fibroids, and 1 with premature rupture of membrane). The participants gave birth vaginally between 37th and 42nd gestational week with average birth weight being 3383.84 g among newborns, including 15 boys and 12 girls. Comparisons of maternal age, gestational age, number of complications, gender of newborns, birth weight and Apgar score group were conducted between the two groups. We only identified different gestational age distributions between AG and NG groups (*P* value = 0.01, *t*-test).

**Table 1:** Demographic and clinical characteristics of the two groups

Characteristics	AG = 12	NG = 15	*P* value
Maternal age, years, (mean, SD)	32.67 ± 3.73	32.07 ± 2.70	0.64
Gestational age, weeks, (mean, SD)	39.58 ± 0.64	38.80 ± 0.77	0.01*
With complications (without drug intervention)	5	5	0.96
Gender of infants (Male)	9	6	0.15
Birth weight, g, (mean, SD)	3485.83 ± 345.42	3296.43 ± 235.98	0.11
Apgar score, (mean, SD)	10	10	1

AG: pregnant women with penicillin allergy; NG: non-allergic pregnant women

### Overall community structure of vaginal microbiome

Of 1,652,922 high quality paired-end reads were generated from 27 samples. An average of 61,219 (range: 60,623–62,058) sequences per sample was recovered for downstream analysis. A total of 737,154 sequences (average: 61,430) were obtained from penicillin allergic women for analysis, while 915,768 sequences (average: 61,051) were obtained from non-allergic pregnant women.

**Fig. 1:**
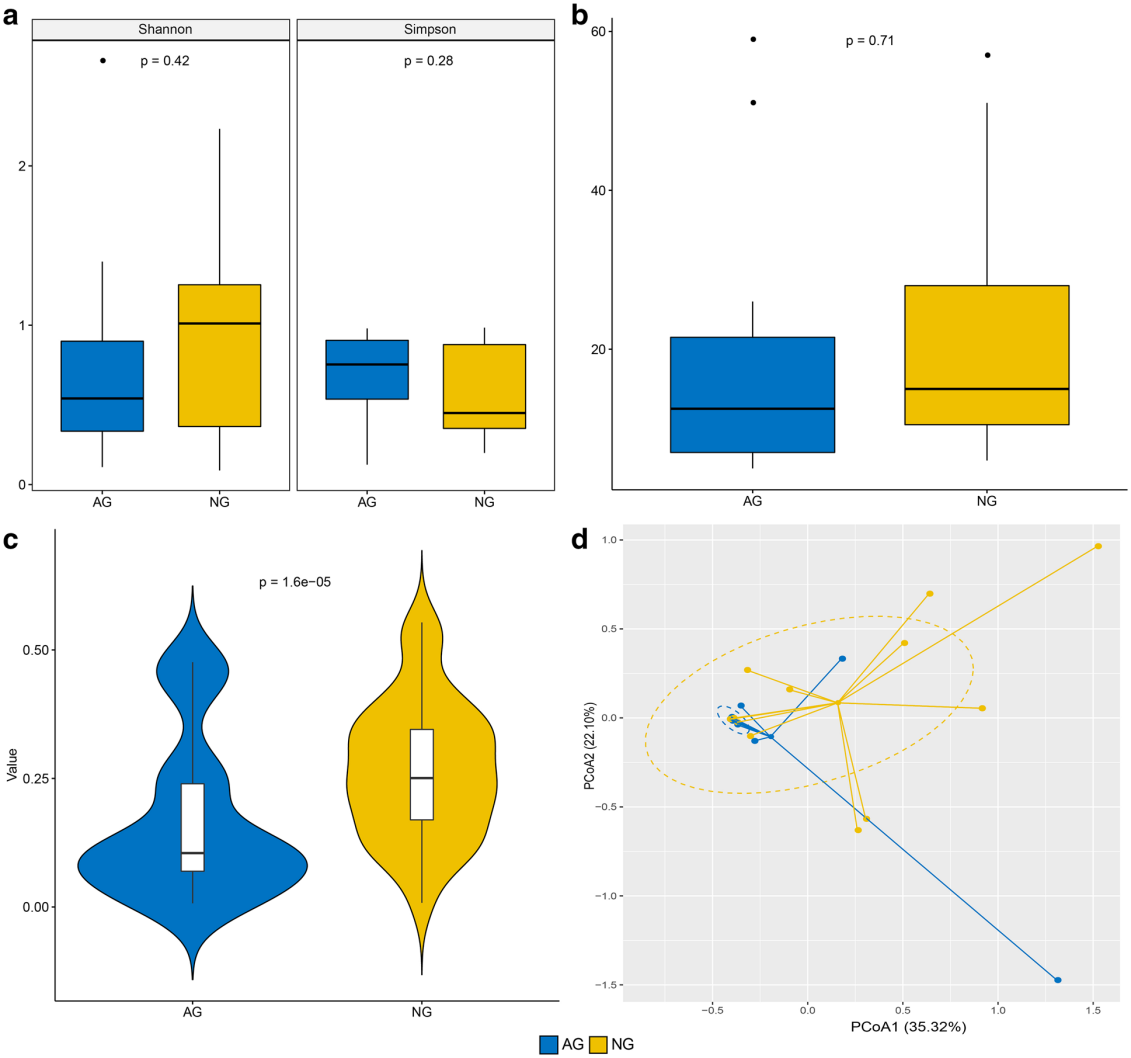
Diversity and principal coordinates analysis (PCoA) plot in microbiota community structure of allergic (AG) and non-allergic women (NG). **a** Distribution of Shannon and Simpson indices in pregnant women with and without antibiotic allergy. **b** Comparison of observed OTU number between the two groups. **c** Difference of weighted UniFrac value between the two groups. **d** PCoA plot based on bacterial abundances

Microbial community diversity was demonstrated in this study using Shannon, Simpson indices and observed OTU number. Based on the microbial distributions, the average value of Shannon index was 0.76 ± 0.71 (mean ± SD) and 0.98 ± 0.63 in allergic and non-allergic women, respectively (*P* = 0.42, *t*-test, Fig. 1a). The Simpson index also presented without significant discrepancy, averaging 0.69 ± 0.24 in penicillin allergic group, while 0.58 ± 0.28 in non-allergic group (*P* = 0.28, *t*-test, Fig. 1a). Moreover, the average value of observed OTU number in non-allergic group (21.93 ± 15.84) was a little higher than allergic group (19.42 ± 17.90) (*P *= 0.71, *t*-test, Fig. 1b). Whereas, the weighted UniFrac value of penicillin allergic women (0.16 ± 0.15) was significantly lower than that of non-allergic women (0.26 ± 0.13) with *P* value less than 0.01 (*t*-test, Fig. 1c). Then principal coordinates analysis was implemented on the bacterial abundances to compare the overall vaginal microbiome composition between AG and NG groups. Figure 1d showed that most subjects between the two different groups overlapped, indicated a similar structure. Subjects with penicillin allergy clustered more tightly together (expect one sample) than the non-allergic subjects, consisted with the result of Fig. 1c.

### Microbial community structure and different microbes in pregnant women with and without penicillin allergy

**Fig. 2:**
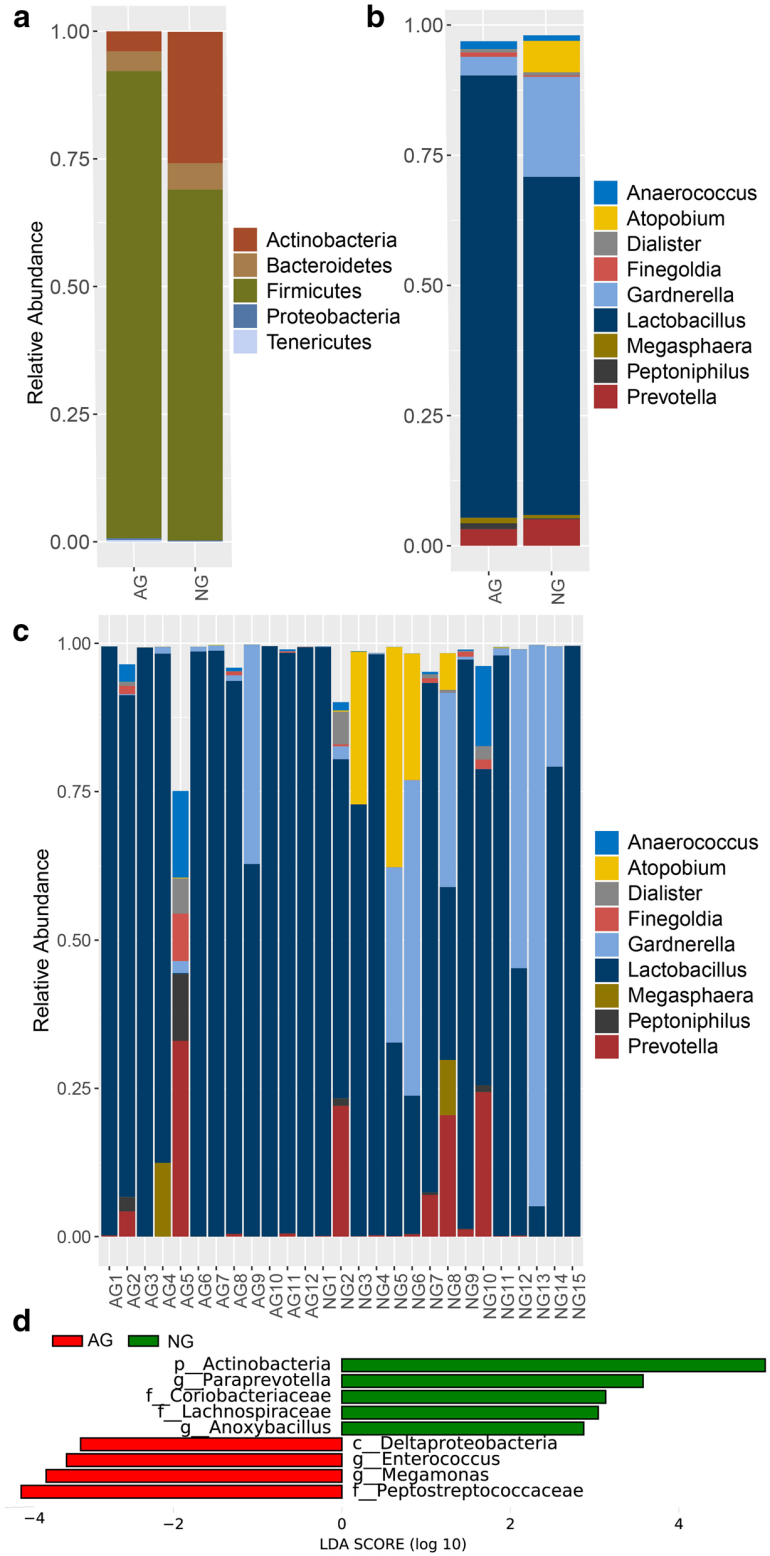
Microbial profiles of vaginal swab samples and differential microbes between allergic (AG) and non-allergic women (NG) groups. **a** Relative proportions of the dominant phylum. **b** Relative abundances of the dominant genus. **c** Relative abundance of each subject at the genus level. **d** Enriched microbes in pregnant women with and without penicillin allergy detected by LEfSe

The relative abundances of the main phyla for AG and NG groups were shown in Fig. 2a, which included *Firmicutes, Actinobacteria, Bacteroidetes, Proteobacteria* and *Tenericutes*. Among these phyla, *Firmicutes* was the predominant bacterium both in AG and NG groups, accounting for 91.51% ± 16.4% and 68.75% ± 31.63%, respectively, which decreased in NG group. Compared to AG group, the average relative abundances of *Actinobacteria* (3.9% ± 10.49% versus 25.69% ± 32.46%) and *Bacteroidetes* (3.87% ± 11.65% versus 5.16% ± 9.16%) increased in NG group.

The main genera showed in the enrolled subjects were *Lactobacillus*, *Gardnerella*, *Prevotella*, *Atopobium*, *Anaerococcus*, *Dialister*, *Finegoldia*, *Megasphaera* and *Peptoniphilus* (Fig. 2b). The proportion of *Lactobacillus* accounted for 84.94% ± 28.79% in AG group, higher than NG group (64.93% ± 32.11%). Instead, the average proportions of *Atopobium* (0.02% ± 0.04% versus 6.04% ± 11.89%), *Gardnerella* (3.57% ± 10.52% versus 19.20% ± 28.65%) and *Prevotella* (3.21% ± 9.46% versus 5.09% ± 9.11%) increased in NG group compared to the pregnant women with penicillin allergy. The relative abundance of each participant at the genus level was shown in Fig. 2c. Most of them dominated by *Lactobacillus*, and a few dominated by *Gardnerella, Atopobium* or *Prevotella*.

Through LEfSe analysis, a total of 9 differentially enriched bacterial colonizers with LDA score > 2 (4 in allergic women and 5 in non-allergic pregnant women) were identified (Fig. 2d). Phylum *Actinobacteria* (25.69% ± 32.46%, LDA = 5.02, *P* = 0.032), families *Coriobacteriaceae* (6.09% ± 11.90%, LDA = 3.13, *P* = 0.034) and *Lachnospiraceae* (0.03% ± 0.04%, LDA = 3.04, *P* = 0.045), genera *Paraprevotella* (0.001% ± 0.0018%, LDA = 3.57, *P* = 0.016) and *Anoxybacillus* (0.028% ± 0.061%, LDA = 2.87, *P* = 0.002) were enriched in non-allergic pregnant women. Abundances of class *Deltaproteobacteria* (0.0018% ± 0.0028%, LDA = 3.10, *P* = 0.034), family *Peptostreptococcaceae* (0.002% ± 0.0025%, LDA = 3.81, *P* = 0.044), genera *Enterococcus* (0.0027% ± 0.0029%, LDA = 3.27, *P* = 0.015) and *Megamonas* (0.0007% ± 0.0016%, LDA = 3.51, *P* = 0.044) were enriched in penicillin allergic women.

### AG and NG groups harbor distinctive vaginal microbial co-abundance networks

**Fig. 3:**
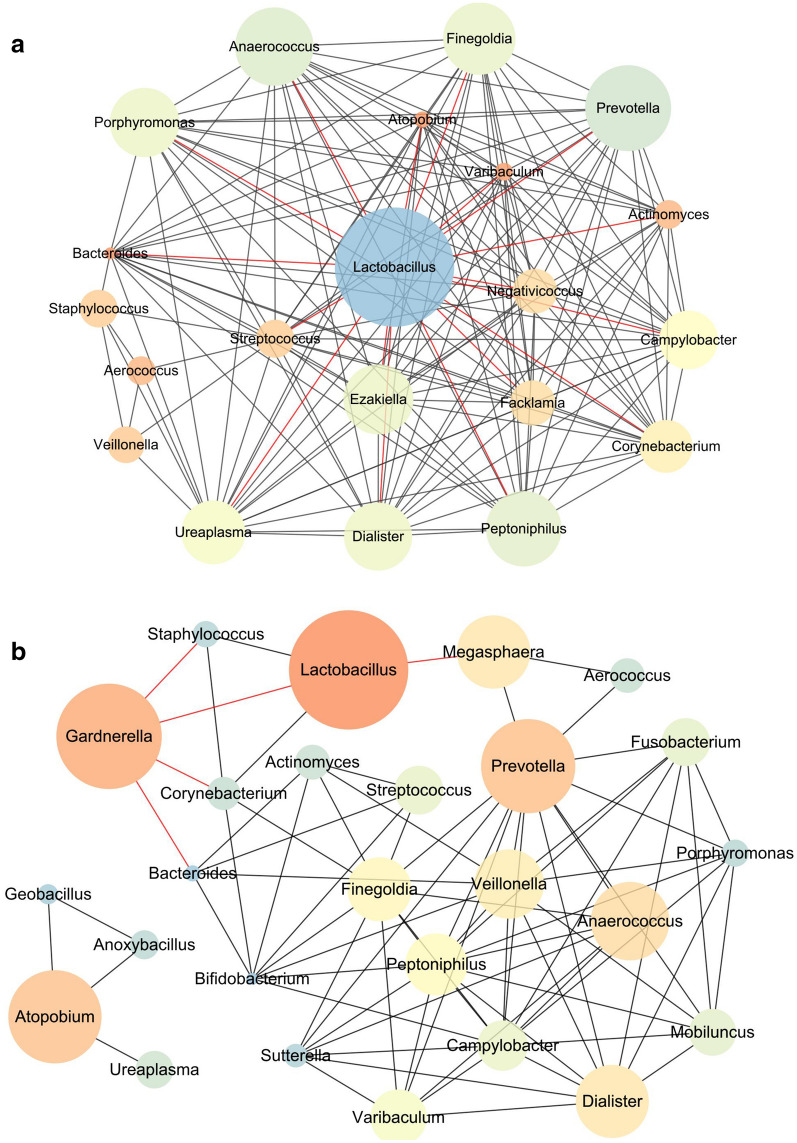
Vaginal microbial networks in penicillin allergic (AG) and non-allergic (NG) pregnant women at the genus level. The correlation analysis among vaginal bacteria was executed, and the relationships with *r* value higher than 0.45 or lower than − 0.35 were kept. The red and black edges represented the negative and positive correlation, respectively. The diameter of the spots was proportional to the relative abundance. **a** Co-abundance network in pregnant women with penicillin allergy. **b** Co-abundance network in non-allergic pregnant women

The co-abundance microbial networks for penicillin allergic and non-allergic women were inferred based on the Pearson index at the genus level (Fig. 3). From the nodes and edges’ perspective, there were many differences in co-abundance networks between AG and NG groups. In penicillin allergic women, 21 genera were observed in the co-abundance network, and all genera were correlated with each other. The results also showed that only *Lactobacillus* was negatively correlated with the other genera (Fig. 3a). However, 26 genera showed in the co-abundance network of non-allergic women. *Lactobacillus* was negatively correlated with *Gardnerella* and *Megasphaere*, and positively correlated with *Staphylococcus* and *Corynebacterium* (Fig. 3b). *Gardnerella* was negatively correlated with *Bacteroides*, *Corynebacterium* and *Staphylococcus*. Enriched genus *Anoxybacillus* was positively correlated with *Atopobium* and *Geobacillus* (Fig. 3b).

## Discussion

This study focused on the vaginal microbial structure of pregnant women with penicillin allergy and its comparison with non-allergic women in China. The abundant phyla of vaginal microbiome in this study were *Firmicutes, Actinobacteria* and *Bacteroidetes*. At the genus level, *Lactobacillus* was the predominant bacteria, in agreement with the previous studies [5, 18], for *Lactobacillus* species play key roles in preventing the occurrence of vaginal disorders [10]. Five bacterial communities have been reported in vaginal microbiome [30, 39], four of them dominated by *Lactobacillus iners*, *L. crispatus*, *L. gasseri* and *L. jensenii* separately, whereas the fifth has lower proportions of lactic acid bacteria and dominated by complex microbial communities of *Gardnerella*, *Atopobium*, *Dialister*, *Peptoniphilus*, *Lachnospiraceae*, *Anaerococcus* and *Prevotella* members [30, 39]. In this study, 21 subjects dominated by genus *Lactobacillus*, and the other 6 subjects dominated by genera *Gardnerella, Atopobium* or *Prevotella*. In order to identify lower taxonomic level than genus in this study, metagenomic sequencing should be conducted in further study.

Microbiome of body is well known to modulate the immune system [42]. Intestinal microbiome plays a key role in balancing the activities of Th1 and Th2 cells to regulate responses to different antigens, and the microbiome in the lung has been proved to be important in the balance between Th2 and Th17 patterns [32]. Notably, the biodiversity hypothesis is proposed to explain the relationships between microbiome and allergy [32], as reducing exposure to microorganisms may affect the developmental mechanism of immunologic tolerance. Evidences indicate that low diversity of microbiota is associated with high risk of developing allergy [19]. In consistent with this hypothesis, both Shannon index value and observed OTU number were lower in allergic women than that of the non-allergic women in this study. Additionally, LEfSe analysis in this study discovered that phylum *Actinobacteria,* families *Coriobacteriaceae* and *Lachnospiraceae*, genera *Paraprevotella* and *Anoxybacillus* significantly decreased, whereas class *Deltaproteobacteria*, family *Peptostreptococcaceae*, genera *Enterococcus* and *Megamonas* significantly increased in penicillin allergic women. In consistent with previous studies, the decrease of *Actinobacteria* is associated with asthma and food allergy [22, 24]. Enriched class *Deltaproteobacteria* is related to allergy in mice [21], and the relative proportion of family *Peptostreptococcaceae* is correlated positively with total serum IgG and IgE levels that relate to allergy [12]. Moreover, *Lachnospiraceae* has been reported to implicate in protection against food allergy through producing acetate, butyrate and propionate to modulate the immune system by inducing Treg cells, DCs precursors and IL-10 production [17, 29], while a decrease trend was observed in allergic women in this study. Low abundances of *Coriobacteriaceae* and *Anoxybacillus* are associated with food allergy in children [8, 14]. All these studies indicate that shifts of vaginal microbiota observed in this study may be closely related to allergy. However, the effect of vaginal microbiome on the Th1, Th2 or Th17 cells is not clear due to the limited data of helper T cell subsets in this study.

Penicillin allergy is more prevalent in families with allergic history [4, 16, 25]. An inheritance of penicillin allergy between parents and infants may exist. Maternal microbiota during pregnancy has been found to play a key role in preventing an allergy-prone immune phenotype in the offspring, and the microbiota community structure in the first few months of life is associated with allergy development in later childhood [45]. As the largest source of the initial gut microbiota in the newborns, the alterations of vaginal microbiome in allergic women may impact the neonatal microbial colonization, and make them more susceptible to allergy. In this study, a slightly lower microbial diversity and significantly lower beta diversity in pregnant women with penicillin allergy than non-allergy was observed. In accordance with previous studies that a lower gut microbial diversity also existed in infants with asthma or allergy [1, 2, 23]. Furthermore, in the co-abundance network of non-allergic pregnant women, *Staphylococcus*, *Corynebacterium* and *Lactobacillus* were positively correlated with each other, and negatively correlated with *Gardnerella*, while not in penicillin allergic women. Bacterial colonization starting with *Staphylococcus* or *Corynebacterium* more commonly occur in healthy infant [43], compare to allergic infant. *Lactobacillus* species are usually taken as probiotics, their increased proportions promote a long-term protective effect against food allergy in the offspring [10, 11]. All these indicate that microbial transmission from mother to children may be related to familial aggregation of penicillin allergy. But the effect of vaginal microbiome of penicillin allergic women on neonate could not be defined due to limited data. In the near further, neonatal data and animal experiments should be conducted to elucidate the effects of vaginal microbiome and its relationship with familial aggregation of penicillin allergy.

Furthermore, the small sample size, subjects with complications and all subjects were recruited from one hospital, which would affect the accuracy of the discoveries in this study. A large sample size and a multicenter clinical study should be considered to address above issues. Previous studies demonstrated that microbiome could modulate the immune response through interactions with both the innate and acquired branches of the immune system [29, 31]. In this study, a different vaginal microbial community structure was observed in the penicillin allergic pregnant women, but the underlying mechanism was not clear due to limited data. So the data representing responses of immune system should be collected and animal experiment research would be needed to elucidate the mechanism. Additionally, different genetic predispositions to polarize responses to different antigens in opposite directions were observed in mice [32], which should also be carefully considered in the further study.

In conclusion, many differences existed in the vaginal microbiol community structure of pregnant women with penicillin allergy, and a different co-abundance network was also observed, compared to the vaginal microbiome of non-allergic women.

## Data Availability

16S rRNA sequence data and metadata for each sample have been deposited in the National Center for Biotechnology Information Sequence Read Archive (NCBI SRA BioProject ID PRJNA595036).
